# Salivary and gingival crevicular fluid histatin in periodontal health and disease

**DOI:** 10.4317/jced.51106

**Published:** 2013-10-01

**Authors:** Smruti J. Bhadbhade, Anirudh B. Acharya, Srinath L. Thakur

**Affiliations:** 1 Private Consultant. B105, Sarala Roses, Baner Road, Pune, Maharashtra, India; 2 Professor. SDM College of Dental Sciences and Hospital. Sattur, Dharwad, Karnataka, India; 3 Professor and Head. SDM College of Dental Sciences and Hospital. Sattur, Dharwad, Karnataka, India

## Abstract

Objectives: Histatin, with its anti bacterial, anti protease, and wound closure stimulating property might influence the pathogenesis of periodontal disease. This study assessed the presence of histatin in gingival crevicular fluid (GCF); the levels of salivary and GCF histatin in periodontal disease. Material and methods: It was a cross sectional study that included systemically healthy forty five subjects (22 males and 23 females) between the age group of 20 to 45 years. Based on Gingival Index (Loe and Silness ,1963) and Russell’s Periodontal Index they were grouped as 15 healthy (Group 1), 15 gingivitis (Group 2), and 15 periodontitis (Group 3) subjects. Whole pooled unstimulated saliva was collected by asking the patient to spit in a sterile container and GCF samples were collected using a micropipette from all the subjects. Histatin levels were assessed using Enzyme Linked Immunosorbent Assay (ELISA). The intergroup comparison was done by ANOVA and Mann Whitney U Test was done for pair wise comparison. Results: The results of this study show that histatin is present in saliva and gingival crevicular fluid. When the salivary histatin levels were compared it was found that the levels of histatin increase from health to periodontitis but the levels of histatin in the gingival crevicular fluid and saliva had no correlation with severity of periodontal disease as there was no statistically significant difference between the three groups. Conclusions: It can be concluded that histatin cannot be used as a potential marker of periodontal disease.

** Key words:**Periodontal disease, histatin, gingival crevicular fluid, saliva, gingival index, periodontal index, enzyme linked immunosorbent assay.

## Introduction

Histatins are a group of small, cationic histidine-rich peptides secreted by human salivary glands. A number of different biological functions have been ascribed to the histatins: It is involved in the formation of acquired enamel pellicle (1), binds to the *Porphyromonas gingivalis* and inhibits its trypsin like activity (2). It has anti fungal and bactericidal activity (3,4). It prevents coaggregation, inhibits bacterial proteases at physiological concentration and has wound closure stimulating property (5-7). It has shown to reduce the clinical signs of gingival inflammation and plaque formation (8). These characteristics of histatins are a good reason to investigate any possible role played by them in the periodontal disease (P.D.).

In order to find the role played by histatins in the pathogenesis of periodontol disease their presence in the gingival crevicular fluid (GCF) needs to be investigated and its relation to periodontal health and disease evaluated.

Considering the strong analogy that exists between saliva and gingival crevicular fluid (GCF) as potential sources of PD markers and the already known presence of histatin in saliva (9), the possible presence of histatin in GCF and its role in PD was considered. Hence this study aimed to evaluate the presence of histatin in GCF, assess the levels of salivary and GCF histatin in periodontal health and disease.

## Material and Methods

Forty five subjects (23 females and 22 males), in the age range 20 to 45 years, were recruited for the study that was conducted in June and July of 2010, at the Sri Dharmasthala Manjunatheshwara College of Dental Sciences and Hospital, Dharwad, India. Informed written consent was obtained from all subjects before starting the study. The ethical clearance was obtained from this institution. Three groups with 15 subjects each were designated as Group 1 (clinically healthy), based on Gingival Index (10) score of 2 and 3, Group 2 (gingivitis), based on Russell’s Periodontal Index (1967) (11) score of 6 and 8 as Group 3 (chronic generalized periodontitis) respectively. Group 3 included the chronic generalized periodontitis group with moderate to severe type.

Subject inclusion criteria:

1. Healthy subjects and patients with varying degree of periodontal disease.(Gingivitis and chronic generalized periodontitis).

2. Good general health.

3. No periodontal therapy in the past 6 months.

Subject exclusion criteria:

1. Patients with any systemic disorder like diabetes mellitus, hypertension etc.

2. Patients with a known disorder of the salivary gland.

3. Presence of any disorder that might alter the immune system.

4. Patients on any medication that might alter the salivary flow rate or composition. E.g. Beta blockers.

5. Patients who had been administered antibiotics in the past 6 months.

6. Patients consuming tobacco in any form were excluded from the study as the relation of tobacco and histatin levels is not ascertained till date.

A dental and medical history was compiled for all subjects with an oral examination, including caries assessments. Gingival Index and Russell’s Periodontal Index scores were collected for each subject. The same investigator performed all data collection and examinations.

- Determination of Salivary and GCF Histatin:

* Collection of saliva

Participants were asked to refrain from drinking or eating half an hour before collection of saliva sample.

Both test and control subjects reported to the hospital between 10.00 am and 2.00 pm. One millilitre (ml) of unstimulated pooled whole saliva was collected by asking the patient to spit in a sterile container. The patients were told to avoid spitting forcefully in order to avoiding any possible contamination. The saliva samples were centrifuged at 3000 × g for 20 minutes, and the clear supernatant was stored at – 70°C until assay was performed.

* Collection of GCF

Multiple test sites were dried and isolated with cotton rolls to prevent any contamination from saliva and blood. Prior to GCF sampling, supragingival calculus was removed using sterile curette. A standard volume of 2 μl was collected extracrevicularly using a calibrated, volumetric, microcapillary pipette measuring 1 to5 μl with a plunger for 5 to 30 minutes. Pooled volume of GCF was collected for healthy subjects where as for gingivitis and periodontitis site specific sampling was followed. Samples were collected from sites exhibiting severe inflammation with Gingival Index score of 2 and 3 for gingivitis group (Group 2) and sites exhibiting a probing depth of >5 mm for periodontitis group (Group 3). On visual examination, test sites which did not express any volume of GCF and micropipettes contaminated with blood and saliva were not included in the study. The GCF obtained was stored at – 80°C until the assay was performed.

- Histatin determination:

It was determined by using a sandwich ELISA technique as described by Jensen et al (12). Saliva was diluted to 1:100 dilution by addition of 4 µl of saliva to 400 µl of phosphate buffered saline. 50 µl of this sample was added to each well.

Gingival crevicular fluid was also diluted to 1: 100 where 2 µl of GCF was added to 200 µl of phosphate buffe-red saline and 50 µl of this sample was added to each well. PBS was used as blanks. Histatin 5 $ (range, from 31.2 ng/mL to 2 µg/mL) were used as standards. The absorbance was read at 450nm in a microtitre plate reader.

$sc-28111 Santa Cruz®, USA

- Statistical evaluation:

The data collected was entered in Microsoft Office Excel Format and statistical analysis was done using SPSS software.§ One-way ANOVA was done to test the significant difference between the groups. Statistical significance was established at P <0.05.

## Results

All the values are expressed as optical density and the p value <0.05 is considered to be statistically significant.

All the samples in each group showed the presence of histatin. ( Table 1) The highest concentration being in group 3 for salivary histatin. The histatin readings range from 0.510 in healthy subjects to 0.865 in subjects with periodontitis. The mean for healthy subjects was 0.667, for gingivitis was 0.7093 and for periodontitis was 0.7253. All the gingival crevicular fluid samples showed the presence of histatin. ( Table 2) The histatin readings for gingival crevicular fluid did not show any ordered rise or fall with level of periodontal disease. These values show that there is a negligible difference in histatin levels in the gingival crevicular fluid with increase in severity of disease, i.e., from health to gingivitis and to periodontitis. With mean values for health, gingivitis and periodontitis being 0.6381,0.6334 and 0.6654 respectively.

**Table 1:**
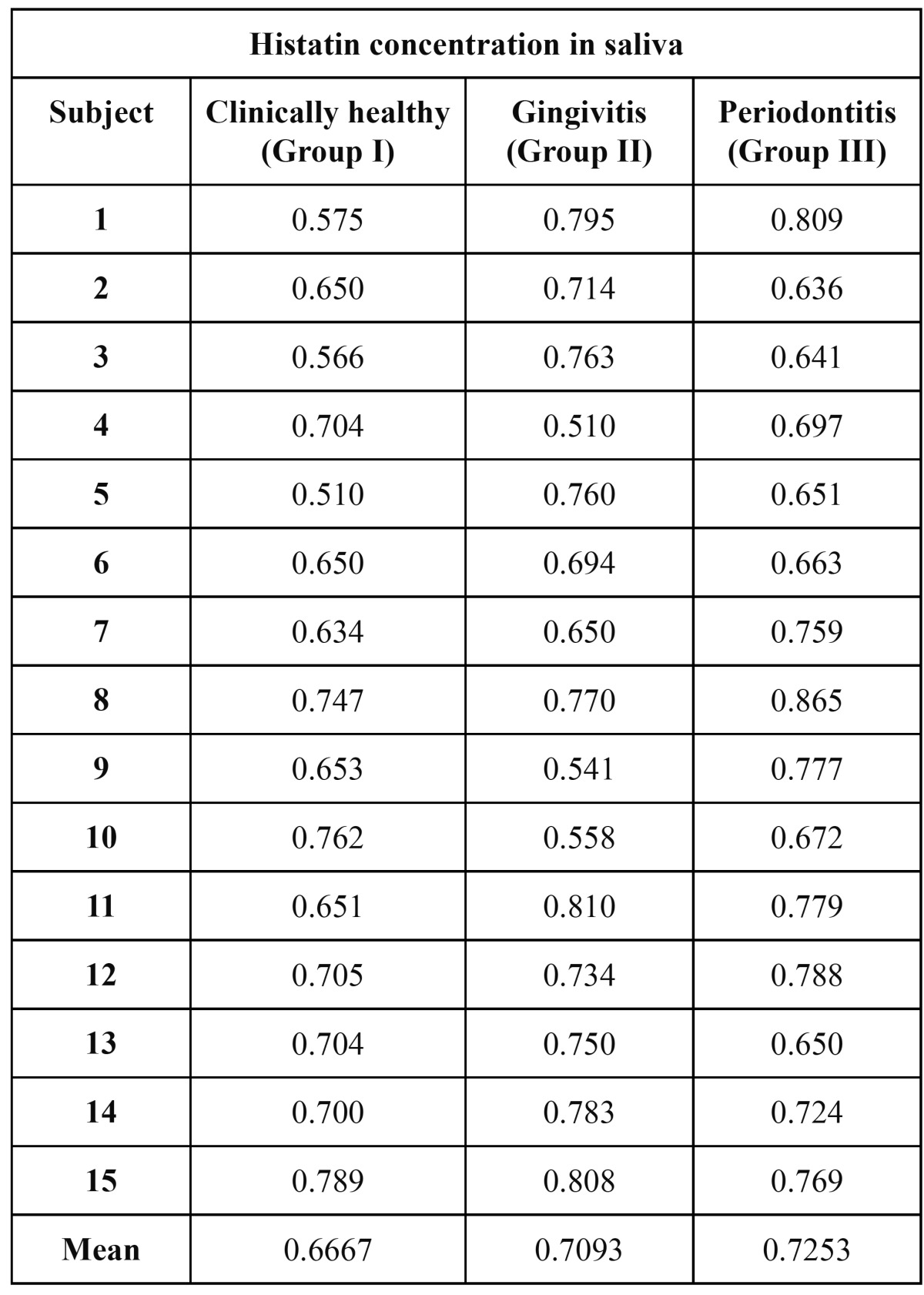
Concentrations of histatin in saliva of Group I, Group II and Group III.

**Table 2:**
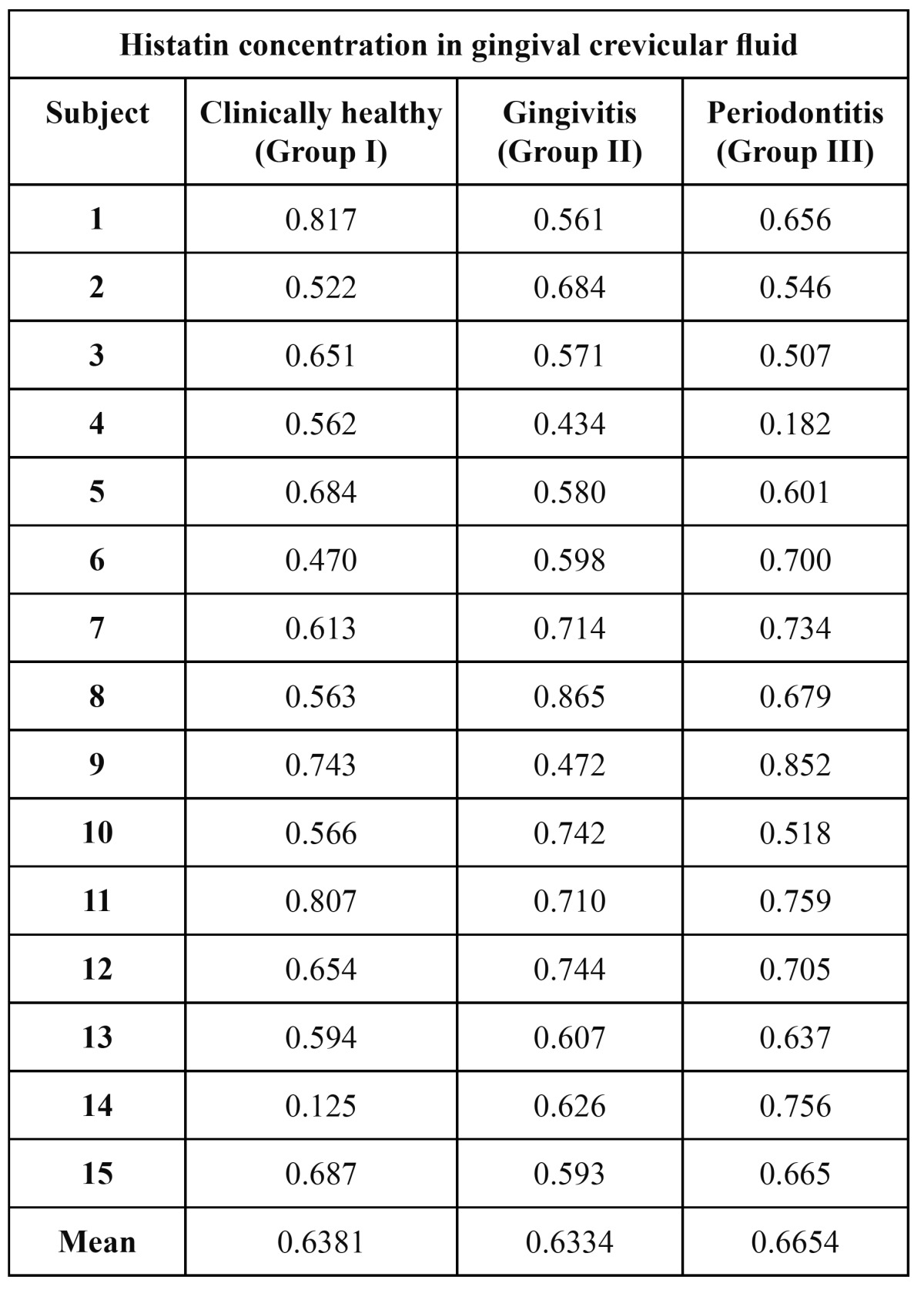
Concentration of histatin in gingival crevicular fluid of Group I, Group II and Group III.

One-way ANOVA was used to test the significant difference between the groups and a no significance differen-ce in the mean concentration of histatin was observed in both saliva and GCF ( Table 3,  Table 4). Mann Whitney U test was done for the inter group comparison.  Table 3,  Table 4). The p values for Salivary histatin in intergroup comparison were 0.0971, 0.0815, 0.9669 and none were statistically significant. The p values with GCF histatin were 0.8035,0.3095 and 0.4186 which also failed to show statistical significance.

**Table 3:**
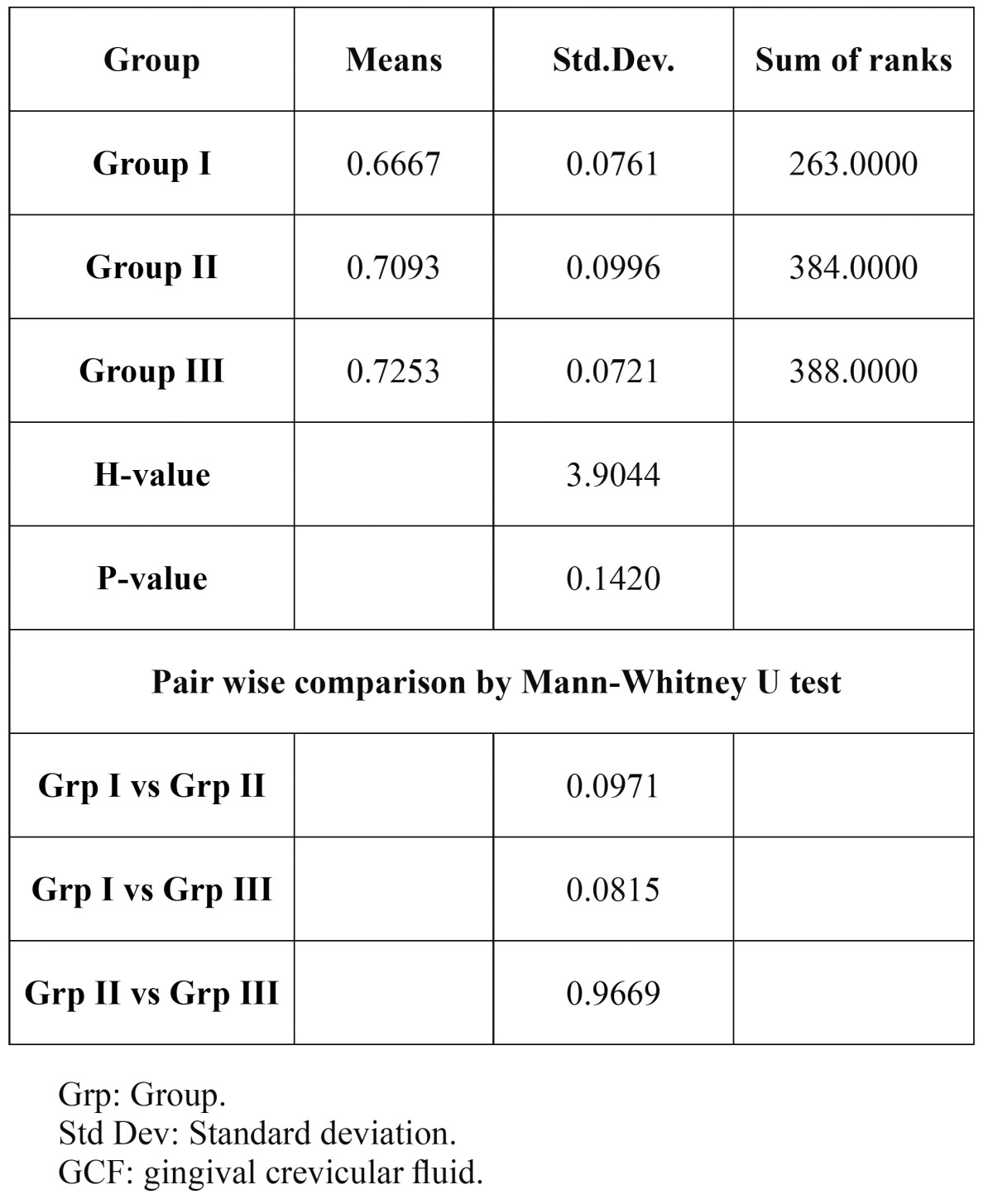
Comparison of the GCF histatin levels in the three groups by Kruskal Walllis ANOVA.

**Table 4:**
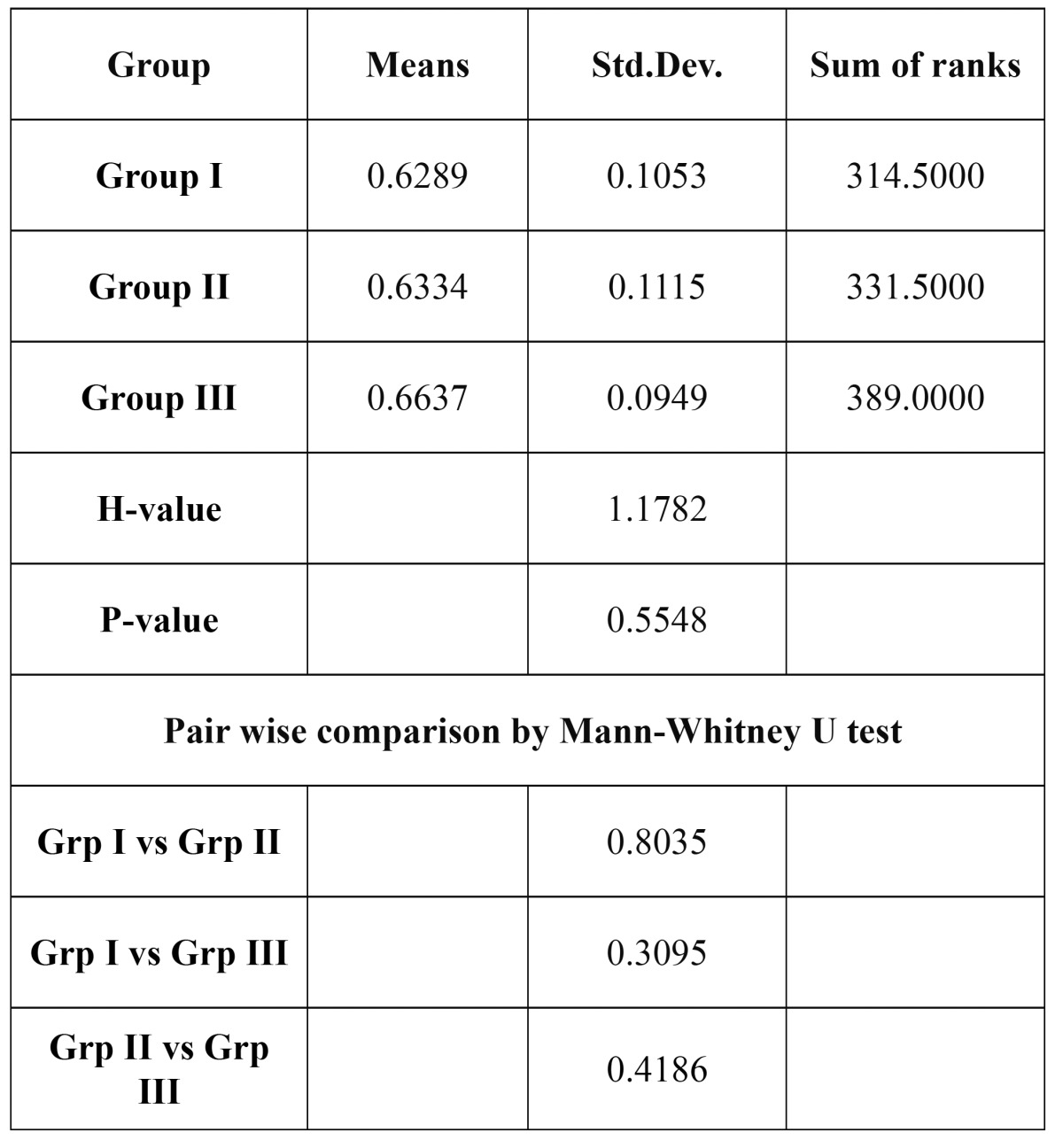
Comparison of the salivary histatin levels in the three groups by Kruskal Walllis ANOVA.

The results suggest that the levels of salivary and GCF histatin did increase slightly from health to disease but it was not statistically significant.

§ SPSS statistical package (PC version 7.0), SPSS, Chicago,IL.

## Discussion

With respect to the subject selection, systemically healthy subjects were chosen as not much literature was available as to which systemic conditions can influence histatin secretion. Moreover, any disorder of salivary glands or the immune system is bound to alter histatin secretion and therefore, the subjects with either of the above were excluded. As β blockers tend to cause decreased histatin secretion, subjects on these medications in particular and any other medication in general were not recruited (12). Equal number of males and females were recruited to prevent bias due to gender but it has been reported that there is no alteration in histatin secretion with respect to sex (13). Individuals within the age range of 20-45 years were chosen as older individuals have a higher incidence of xerostomia that might directly affect salivary histatin levels and indirectly affect the study due to increased difficulty in sampling (14). Also the possibility of older subjects being on some kind of medications and most commonly anti- hypertensive medications is very high.

Time of sample collection was chosen to be around 10.00 am to 2.00 pm as that is the time of the day when the histatin secretion peaks in both the submandibular and parotid glands respectively (13).

In order to correlate the levels of salivary histatin to periodontal disease whole saliva was collected from the subjects. It has been stated in the literature that the levels of histatin in glandular secretions is higher than in the whole saliva (15). The reduction of histatins in whole saliva could be due to binding of histatins to hydroxyapaptite and proteolytic effect of oral microflora on histatin (15). It is the whole saliva that contacts and affects the periodontium, hence whole and not glandular saliva was chosen to be sampled. Saliva stimulation, both, gustatory or mechanical results in increase of histatin secretion. But this stimulation occurs only during a limited period of time in the day. So resting saliva was chosen to be sampled (12).

Collection of GCF using microcapillary pipette has been widely used (16-18). It has been shown to be a decei-ving method with respect to sample collection, in clinically healthy subjects due to vary little amounts of gingival crevicular fluid present in health this could be one of the limitations of the study (19). However studies have shown that an average of 10-40µl of GCF can be collected in subjects with Gingival and Plaque index score of 3. Thus, the use of microcapillary pipettes for GCF collection is not a problem for these patients. Also, it has the advantage as it is not plagued by the nonspecific attachment of the analyte to the filter paper (20).

The results of this study show the presence of histatin in saliva of all the samples. The increase in its levels with respect to periodontal disease might was slight and failed to reach a statistically significant value, suggesting that onset and progression of periodontal disease might not have any effect on histatin secretion from the salivary glands. Thus its utility as a salivary biomarker is questionable and also its role in periodontal disease activity i.e., either progression or remission cannot be adequately substantiated.

With respect to the levels of histatin in the gingival crevicular fluid all the samples showed its presence but no statistically significant difference could be found out with respect to the periodontal disease. The origin of histatin in the gingival crevicular fluid could be attributed to the characteristic of it being a transudate/exudates and that histatin has been detected in the serum in previous studies (21). There is no variation in the GCF histatin with respect to increase in severity of periodontal disease. This might be due to the inability of periodontal disease to cause significant change in serum histatin levels that are expected to be reflected in the gingival crevicular fluid. This can be confirmed by further studies to correlate histatin levels in serum and periodontal disease state. Thus failure to detect differences in GCF histatin levels disqualifies histatin as a potential biomarker to supervise periodontal health and disease. Also the present study raises doubts if histatin does have any role to play in pathogenesis of periodontitis.

The results of this study show the presence of histatin in the saliva samples of all the subjects. The method used in this study to assess histatin levels using ELISA is in accordance with the methodology described by Jensen JL in 1994 (12). But in those previous studies of histatin evaluation, glandular salivary histatins and not whole salivary histatins were evaluated. Thus, to the best of knowledge, this is the first study to evaluate the whole salivary histatins using ELISA technique and hence the values cannot be correlated to those of the previous studies.

A mixture of pure histatin 1-12 was not available and since the antihistatin antibodies used were against all the histatins no standard curve could be plotted and the readings could be obtained only as optical density ratio on the ELISA plate reader - though they can still be used to quantify and correlate histatins to periodontal health and disease though only in terms of optical density. This could be considered another shortcoming of the study and further quantification should be done using other methods of histatin detection.

With respect to the histatin levels in gingival crevicular fluid this is the first study to the best of knowledge, to assess the presence and quantify it in GCF though a previous study that quantified peptides in GCF failed to detect histatin (22). So, further studies using other techniques of histatin quantification need to be carried out in order to support or refute the findings of the present study.

The amounts seen in this study cannot be standardized due to the relatively small sample size and further studies with larger sample size and different quantification techniques need to be undertaken.

It can hence be concluded that histatin in gingival crevicular fluid or saliva could not be considered as a potential biomarker of periodontal disease.
